# Physicochemical properties and enzymatic activity of wheat germ extract microencapsulated with spray and freeze drying

**DOI:** 10.1002/fsn3.2104

**Published:** 2021-01-08

**Authors:** Fahimeh Jamdar, Seyed Ali Mortazavi, Mohammad Reza Saiedi Asl, Akram Sharifi

**Affiliations:** ^1^ Department of Food Science and Technology Sabzevar Branch Islamic Azad University Sabzevar Iran; ^2^ Department of Food Science & Technology Faculty of Agriculture Ferdowsi University of Mashhad Mashhad Iran; ^3^ Department of Food Science and Technology Faculty of Industrial and Mechanical Engineering Qazvin Branch Islamic Azad University Qazvin Iran

**Keywords:** DPPH free radical scavenging activity, maltodextrin, microencapsulation, wheat germ, whey protein concentrate

## Abstract

Wheat germ is produced as a by‐product during wheat milling operations and is a relatively inexpensive protein source that, in spite of its exclusive nutritional properties, is mostly used for animal feed formulation and has limited use in the food industry. In this study, wheat germ extract (WGE) was microencapsulated by spray and freeze drying and with different ratios of maltodextrin to whey protein concentrate (M‐W) as the coating material and then physicochemical properties of the microcapsules were evaluated. Results showed decreased moisture content and increased solubility, lipase activity, acid phosphatase activity, and both lipase and acid phosphatase microencapsulation efficiency with increasing M‐W ratios in both drying methods. The M‐W ratios had no significant effects on the DPPH free radical scavenging activity in both methods. With increasing M‐W ratios, particle size decreased and bulk density increased in the spray drying method, while particle size increased and bulk density decreased in the freeze drying method. Spray drying elevated solubility, DPPH free radical scavenging activity, lipase activity, acid phosphatase activity, and both lipase and acid phosphatase microencapsulation efficiency, in comparison with the freeze drying method.

## Graphical abstract

In this study, wheat germ extract was microencapsulated by spray and freeze drying and with different ratios of maltodextrin to whey protein concentrate as the coating material, and then, physicochemical properties and enzymatic activity of the microcapsules were evaluated.

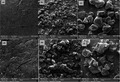

## 1 INTRODUCTION

Since ancient times, cereals have been one of the first known human foods that have always played a critical role in the economy and nutrition of people worldwide, particularly in developing countries. Cereal germ is the richest sources of amino acids, vitamins, and minerals and also contains proper amounts of fiber. They contain a variety of vitamins (A, B, C, D, E, K, and folic acid) and are excellent sources of iron, potassium, calcium, phosphorus, magnesium, and zinc (Almansouri et al., 2001). Among the cereals, wheat (*Triticum aestivum*) represents a foremost cereal product as the main human food crop and livestock feed (Zhang et al., 2019). With the current increasing population growth and raising the knowledge of nutrition quality, wheat germ can be used as a food ingredient. Germ is one of the most attractive and promising source of vegetable functional compounds (Rizzello et al., 2010).

Wheat germ is produced as a by‐product during wheat milling operations and is a relatively inexpensive protein source that, in spite of its exclusive nutritional properties, is mostly used for animal feed formulation and has limited use in the food industry (Rizzello et al., 2010). Wheat germ is a rich source of phytosterols, policosanols, unsaturated fatty acids, protein, lipase, acid phosphatase, flavonoids, B vitamins, dietary fiber, and minerals (Zhu et al., 2006). Wheat germ is the richest known source of plant‐derived vitamin E. Tocopherols are powerful fat‐soluble antioxidants that are effective in preventing cancer, diabetes, hypertension, and Alzheimer's disease. The acceptable percentage of unsaturated fatty acids in wheat germ oil plays an important role in lowering blood cholesterol and treatment of atherosclerosis and heart disease (Dunford, 2009). Antioxidants found in wheat germ include carotenoids, tocopherols, flavonoids, and phenolic acids. Few studies are available on the antioxidants and phenolic compounds of wheat germ. Phenolic compounds are the main antioxidant compounds, and their content is directly proportional to antioxidant activity (Zhu et al., 2011). Enzymes are considerably found in wheat germ, the most important of which include lipase and acid phosphatase. Lipase is widely used in various industries, particularly the food industry, and is commonly applied in the processing of oils and fats, dairy, bakery, etc. The maximum amount of lipase is found in the aleurone and germ cells. (Cara et al., 1992; Elwira et al., 2010). Addition of wheat germ containing valuable compounds and enzymes can be one of the most desirable and simplest methods used to produce products with unique properties.

Recently, plant‐derived bioactive compounds have been widely investigated for their beneficial health outcomes. These metabolites have been receiving a great interest in the last decades for applications in food and pharmaceutical industries due to their antioxidant, antimicrobial, anticancer, antidiabetic, and anti‐inflammatory activities (Shahidi et al., 2020).

Many active compounds, such as antioxidants that are lipophilic, need to be protected from environmental effects (Gibbs et al., 1999). Microencapsulation is a technique by which the sensitive ingredients are packed within a coating or wall material. Microencapsulation technology launched in 1950 and is widely developed today, with wide applications in a variety of pharmaceutical, chemical, food, and printing industries (Augustin et al., 2001). It can envelop a solid, liquid, or gaseous substance within another substance in a very small sealed capsule (Fang & Bhandari, 2010). Microencapsulation can be defined as a process of building a functional barrier between the core and the wall material to avoid chemical and physical reactions and to maintain the functional properties of the core materials (Bakry et al., 2016). Oils and fats, flavoring compounds, oleoresins, vitamins, minerals, color compounds, and enzymes are among the microencapsulated substances in food products (Minemoto et al., 1999). Numerous wall materials or encapsulating agents are available for food application. Gum arabic, maltodextrins of different dextrose equivalent (Bakowska‐Barczaka & Kolodziejczyk, 2011), whey protein powder and mixtures of whey protein and maltodextrin (Bryant & McClements, 1998), pectin and guar gum (Ravichandran et al., 2014) are the most commonly used as wall materials.

The aim of this study was to investigate the effects of type and concentration of coating material and the microencapsulation method on the physicochemical properties and enzymatic activity of microcapsules obtained from WGE.

## 2 MATERIALS AND METHODS

### 2.1 Materials

Wheat germ was purchased from a local flour production factory (Zarrin Khoosheh, Karaj, Iran). Whey protein concentrate (WPC) with 80% protein and maltodextrin with a Dextrose Equivalent (DE) of 9–12 were obtained from Sigma Aldrich (Germany). All the required solutions were procured from Merck, Germany.

### 2.2 Wheat germ extract (WGE)

Wheat germ (30 g) was mixed with 450 ml of distilled water, heated in a waterbath (WNB22‐MEMMERT, Germany) at 60°C for 15 min, and centrifuged (Z36HK‐HERML, Germany) at 4500 rpm for 20 min. The supernatant was collected in dark vessels and stored at −18°C in a freezer (RR30 & RZ30‐SAMAUNG, South Korea) until further tests and the microencapsulation process (Mohamed et al., 2015).

### 2.3 Microencapsulation of WGE

#### 2.3.1 Microencapsulation of WGE by the spray dryer

According to the results of previous studies, maltodextrin and WPC solutions were used to prepare the wall at concentrations of 20% and then placed on a magnetic stirrer (LT108, V. 220, HZ. 50, Iran) at 4,500 rpm for 10 min. To produce a stable emulsion, equal amounts of maltodextrin and WPC (M‐W) were mixed with 1:3, 2:2, and 3:1 ratios (w/w) to reach a total solid content of 40% w/w. WGE was then added to form an optimum core (a core to wall ratio of 1 to 8) and placed on a magnetic stirrer at 4,500 rpm to be mixed for 10 min. Samples were transferred to a spray dryer (B 290, Buchi Laboratoriums‐Technik, Switzerland) at incoming and outgoing temperatures of 150 and 85°C, respectively, with an air flow rate of 35 m^3^/h. Dried samples were collected in dark vials and stored in a freezer (RR30 & RZ30‐ SAMAUNG, South Korea) (Simon‐Brown et al., 2015; Sing et al., 2015).

#### 2.3.2 Microencapsulation of WGE by the freeze dryer

Maltodextrin and WPC solutions were prepared at 20% concentrations for wall preparation and placed on a magnetic stirrer at 90 rpm overnight. Maltodextrin and WPC were mixed with 1:3, 2:2, and 3:1 (w/w) ratios to achieve a total solid content of 40% w/w to form a stable emulsion. WGE was then added to form a desired core (a core to wall ratio of 1 to 8) and stirred on a magnetic stirrer at 5,000 rpm to be mixed for 5 min. The samples were transferred to a freezer at −70°C and then placed in a freeze dryer (Operon FDB‐5503, South Korea) at −55°C with a pressure of 0.15 mm Hg for 48 hr. The obtained powder was kept in dark vials in a freezer until the time of being tested (Basak et al., 2015; Ezhilarasia et al., 2013).

### 2.4 Physicochemical properties of the microcapsules

#### 2.4.1 Moisture content

Moisture content of the powder was determined according to the AOAC method (AOAC, 2005).

#### 2.4.2 Solubility

The solubility percentage of the powders was determined according to the method of Goula and Adamopoulos (2008).

#### 2.4.3 Bulk density

The bulk density of the produced powders was calculated according to the method of Rosenberg et al. (1990).

#### 2.4.4 Particle size

Dispersions of the powders obtained from spray and freeze dryers were prepared in methanol to measure particle size and surface area. Particle size was measured using a particle size analyzer instrument (Oxford, UK) (Jafari et al., 2007).

#### 2.4.5 Morphology of microcapsules by scanning electron microscopy (SEM)

The morphology of the surface of WGE microcapsules of obtained by spray and freeze drying were examined with a scanning electron microscope (LEO 1450 VP Germany). In order to increase the ability of the specimens to conduct electricity and emit secondary electrons, the sample surfaces were previously covered with a very thin gold layer by Fison sputter coater system.

#### 2.4.6 DPPH free radical scavenging activity

DPPH free radical scavenging activity of the microcapsules was determined by the free radical scavenging ability using DPPH method (Brand‐Williams et al., 1995). To this end, 1.0 g of the product was dissolved in 10 ml of distilled water, and then, 100 µl of the solution was mixed with 3.9 ml of DPPH ethanolic solution. The samples were kept in dark for 40 min. Finally, the absorbance of samples was read at 517 nm using a spectrophotometer (T80, PG Instruments, UK) to calculate the percentage of DPPH free radical scavenging.

#### 2.4.7 Lipase activity

Sodium acetate buffer, magnesium chloride, para nitrophenyl phosphate, and distilled water were added to a known weight of the prepared microcapsules and incubated in waterbath (WNB22‐MEMMERT, Germany) at 37°C for 5 min. Then, potash was added, and the absorbance was read at 405 nm by a spectrophotometer (T80, PG Instruments, UK) and was interpreted as μmol para nitrophenol per minute (Stauffer & Glass, 1966).

#### 2.4.8 Acid phosphatase activity

To determine the acid phosphatase activity, monoolein, olive oil, sodium taurocholate, and distilled water were added to a certain weight of microcapsules and the solution was left to form an emulsion for 5 min. Then, a buffer (pH = 7.8) was added and the sample was transferred to a waterbath (WNB22‐MEMMERT, Germany) at 37°C for 5 min, and incubated for 60 min in incubator (MEMMERT‐IN110PLUS, Germany). Finally, trichloroacetic acid and calcium chloride were added, and after filtration, the absorbance was read at 570 nm by a spectrophotometer (T80, PG Instruments, UK) and was interpreted as μmol glycerol per minute (Stauffer & Glass, 1966).

#### 2.4.9 Microencapsulation efficiency for lipase and acid phosphatase

The microencapsulation efficiency of lipase and acid phosphatase was determined according to the following equation (Anjani et al., 2007):

Microencapsulation efficiency (%) = $\frac{\mathrm{Lipase}\mathrm{or}\mathrm{acid}\mathrm{phosphatase}\mathrm{levels}\mathrm{in}\mathrm{microcapsules}}{\mathrm{Lipase}\mathrm{or}\mathrm{acid}\mathrm{phosphatase}\mathrm{levels}\mathrm{in}\mathrm{WGE}}$× 100

### 2.5 Statistical analysis

All experiments were performed with three replications. Data were analyzed in a two‐factor completely randomized design with SPSS 16 software. Means of data were compared with Duncan's multiple range test at 95% confidence level.

## 3 RESULTS AND DISCUSSION

### 3.1 Chemical properties of wheat germ extract (WGE)

The contents of moisture, dry matter, protein, fat, ash, and antioxidant activity in WGE were 95.830, 4.170, 1.702, 2.521, and 0.129%, respectively. Lipase and acid phosphatase activities were 0.176 μmol para nitrophenol/min and 0.182 μmol glycerol/min, respectively.

### 3.2 Effect of drying method and coating material on moisture content of microencapsulated WGE

**FIGURE 1:**
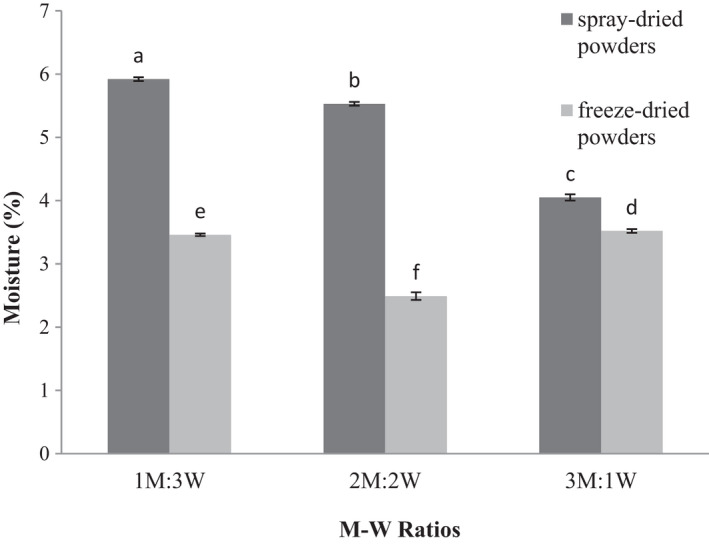
Moisture content of encapsulated wheat germ extract at different ratios of maltodextrin to whey protein concentrate (M‐W)

ANOVA results revealed that increasing the M‐W ratio and drying method significantly affected the moisture content of produced powders (*p* ≤ .05). In spray‐dried powders, the moisture content decreased with increasing M‐W ratios. The highest and the lowest moisture contents were observed in M‐W ratios of 1:3 and 3:1, respectively (Figure 1). Maltodextrin is capable of gel formation and improve moisture retention; therefore, it is used in the food industry as a texturizer (Rosenberg & Sheu, 1996). An increase in the M‐W concentration reduced the water available for evaporation due to the chemical structure of maltodextrin and possibly the difference in the number of water‐bonding groups, thereby reducing the moisture content of the microcapsules.

In the freeze‐dried powders, the highest and the lowest moisture contents were detected in M‐W ratios of 3:1 and 2:2, respectively (Figure 1). An increase in WPC increased the moisture content of the microcapsules, which maybe because WPC was not crystallized upon drying and water removal, resulting in a higher moisture content in the microcapsules with this wall (Selim et al., 2000). The final moisture content of microcapsules is influenced by various factors such as proper capping of storage containers and storage temperature; hence, the observed increase in the 3:1 (M‐W) ratio might have been affected by such factors. Spray‐dried powders contained greater moisture content in all M‐W ratios than freeze‐dried powders, probably due to the differences in the water removal mechanism in each method that affected the final moisture content of microcapsules (Roos, 1995). Frascareli et al. (2012) microencapsulated coffee oil with gum arabic and reported that the moisture content of microcapsules decreased with increasing gum levels (Frascareli et al., 2012).

### 3.3 Effect of drying method and coating material on solubility of microencapsulated WGE

**FIGURE 2:**
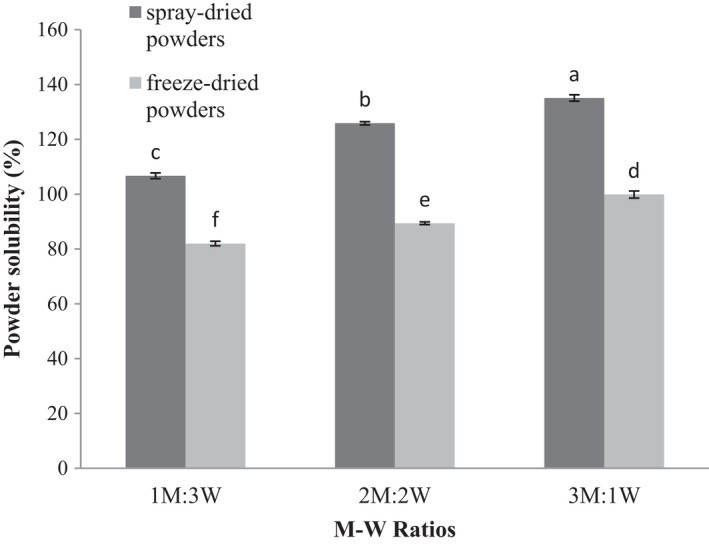
Solubility of encapsulated wheat germ extract at different ratios of maltodextrin to whey protein concentrate (M‐W)

Powder solubility is an important functional property of food powders affecting the powder behavior when it is reconstructed in water (Jayasundera et al., 2011). According to ANOVA results, an increase in the M‐W ratio and drying method had significant effects on the solubility percentage of the produced powders (*p* ≤ .05). An increase in the M‐W ratio led to increases in the solubility percentage of the powders produced by both spray and freeze dryers. The M‐W ratios of 3:1 and 1:3 yielded the highest and the lowest solubility (Figure 2). Important functional properties of maltodextrin include contribution to dispersivity and solubility, control of freezing, prevention of crystallization, and creation of spreadability properties in the product (Marcoa et al., 2013).

High dissolution rates in a wide range of pH, water binding capacity, gelling properties, emulsification ability, and viscosity and texture improvement are among desirable properties of whey proteins for use as coating materials in microencapsulation (Gunasekaran et al., 2007). Therefore, maltodextrin and WPC as microencapsulation agents can interact with WGE, leading to good solubilization of powders obtained from microencapsulated WGE in water (Marcoa et al., 2013).

Spray‐dried encapsulated WGE showed higher solubility in all M‐W ratios than freeze‐dried encapsulated WGE (Figure 2). High‐temperature drying in the spray drying improved the solubility of microencapsulated samples compared to low‐temperature freeze‐drying. Because an increase in the temperature leads to increased particle size, and coarser particles dissolve more easily in the water than finer ones (Walton, 2000). Similarly, Sharifi et al. (2015) showed that the use of maltodextrin in the microencapsulated powder of barberry extract could increase solubility (Sharifi et al., 2015).

### 3.4 Effect of drying method and coating material on bulk density of microencapsulated WGE

**FIGURE 3:**
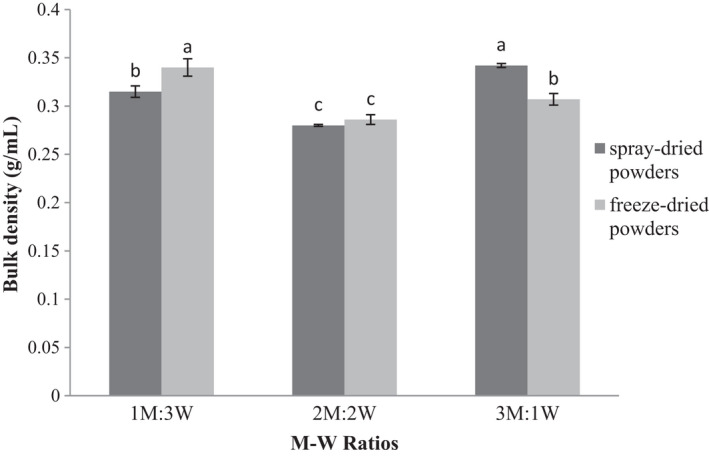
Bulk density of encapsulated wheat germ extract at different ratios of maltodextrin to whey protein concentrate (M‐W)

Bulk density is one of the food properties that depends on the powder size, shape, surface properties, and particle size so that smooth and uniform powders have higher bulk density (Athanasia et al., 2004). The results of ANOVA showed significant effects by increasing M‐W ratios and drying method on the density of produced powders (*p* ≤ .05). An increase in the M‐W ratio raised the density of spray‐dried powders. The highest and the lowest densities were measured in M‐W ratios of 3:1 and 2:2, respectively. Conversely, a decrease in the M‐W ratio elevated the density of freeze‐dried powders, with the highest and lowest densities in the M‐W ratios of 1:3 and 2:2, respectively (Figure 3). The higher bulk density with rising concentrations of wall material can be attributed to the high molecular weight of microcapsules. Materials with high molecular weights are more easily inserted in the interparticle spaces and increase the bulk density with less space (Goula & Adamopoulos, 2008).

The bulk densities of powders obtained by spray and freeze dryers were not significantly different in M‐W ratio of 2:2 and presented the lowest bulk density. This result is mainly due to the similar effects of different carriers, such as maltodextrin and WPC, in reducing particle adhesion, crust formation, increasing porosity, and entrapment of air within particles, leading to reduced particle density (Bazaria & Kumar, 2016). The bulk density of spray‐dried powders in the M‐W ratio of 1:3 was lower than that of freeze‐dried powders in the same ratio. An increase in the temperature raises the porosity, declines wrinkles, and eventually reduces the bulk density of microcapsules (Bazaria & Kumar, 2016). The bulk density of spray‐dryer powders in the M‐W ratio of 3:1 is higher than that of freeze‐dryer powders in the same ratio. The lower porosity of the microcapsules formed by the spray drying method results in less volume occupation than those prepared by freeze drying that elevates the bulk density. This trend is probably due to the effect of increased maltodextrin concentrations relative to WPC with similar dehydration (Zuidam & Shimoni, 2010).

Ferrari et al. (2013) reported that samples containing maltodextrin with gum arabic had greater interparticle space than those prepared with tapioca starch. A greater interparticle space indicates higher amounts of oxygen available for the decomposition reactions leading to rapid loss of coating materials.

### 3.5 Effect of drying method and coating material on particle size of microencapsulated WGE

An increase in the M‐W ratio and drying method significantly affected the particle size of produced powders (*p* ≤ .05), resulting in decreased particle size of spray‐dryer powders. Particle size was uppermost in the M‐W ratio of 1:3, and the smallest particle size was recorded in the M‐W ratio of 3:1 in spray‐dried powders.

**FIGURE 4:**
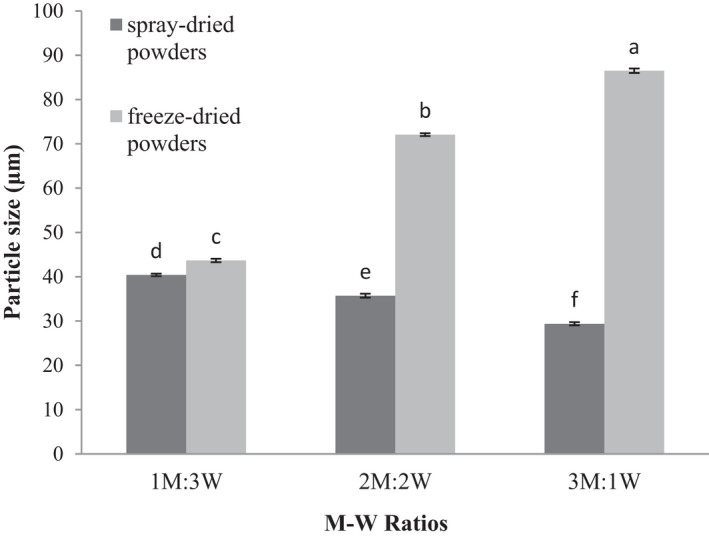
Particle size of encapsulated wheat germ extract at different ratios of maltodextrin to whey protein concentrate (M‐W)

The particle size of the freeze‐dried powders increased with rising the M‐W ratio, with the highest and the lowest particle sizes in M‐W ratios of 3:1 and 1:3, respectively (Figure 4). The particle size of freeze‐dried powders depends largely on the wall material type. Maltodextrin‐containing microcapsules as the wall material produced larger particles than WPC microcapsules. An increase in WPC concentration reduces the particle size diameter, which can be justified by the fact that the WPC emulsifying property is related to hydrophobic particles present therein. High amounts of WPC lead to better emulsification, and extracts are dispersed in the emulsion, yielding smaller particle sizes (Euston et al., 2000). Increased velocity of air flow in spray dryer also reduces the particle size, which in turn raises the drying rate through faster diffusion rates and lower moisture (Simon‐Brown et al., 2015).

Particle size of freeze‐dried powders increased in all M‐W ratios in comparison with that of spray‐dried powders. Initial freezing prior to freeze drying resulted in the accumulation of emulsion droplets and their attachment to each other, leading to a larger final size of microcapsules obtained from this method (Desobry et al., 1997). It was reported that that particles microencapsulated with gum arabic had lower diameters than those microencapsulated with tapioca starch and maltodextrin. Maltodextrin with high degree of hydrolysis also produces smaller particles (Tonon et al., 2010). Decreased particle size of wheat germ oil microencapsulated with different M‐W ratios was observed with increasing WPC concentrations (Basak et al., 2015). A similar result was obtained with microencapsulation of ginger extract by gum arabic and maltodextrin (Simon‐Brown et al., 2015).

### 3.6 Morphology of microcapsules by SEM

**FIGURE 5:**
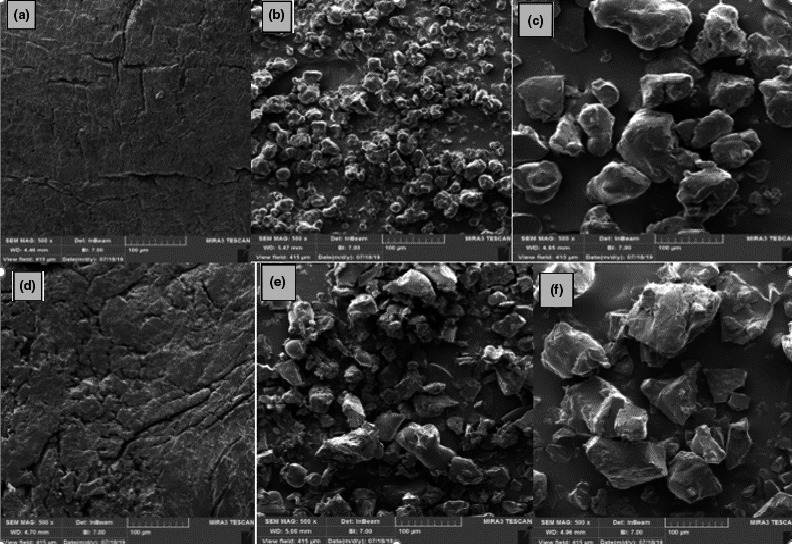
Scanning electron microstructure of spray‐dried and freeze‐dried encapsulated wheat germ extract at different ratios of maltodextrin to whey protein concentrate A) M‐W 3:1, B) M‐W 2:2, C) M‐W 1:3, D) M‐W 3:1, E) M‐W 2:2, and F) M‐W 1:3

Scanning electron micrographs show spray‐dried microcapsules have a spherical structure, and those obtained from freeze drying are of irregular and glassy structure (Figure 5). In spray‐dried microcapsules, the uniformity of shapes improved with an increase in the M‐W ratio, with decreased particle fracture, cracks, and dents on the surface. The incorporation of microencapsulated extract ingredients with carbohydrates (maltodextrin) into the wall improves the drying properties of the wall matrix as a result of an enhancement in the formation of a dry crust around dry emulsion droplets (Sheu & Rosenberg, 1995).

An increase in the M‐W ratio led to elevated amounts of dents and cracks and formation of indentations in freeze‐dried microcapsules, indicating the occurrence of shrinkage during the drying process (Simon‐Brown et al., 2015).

WPC has a profound effect on the surface structure and morphology of microcapsules. Researches have shown that the structure of microcapsules is affected by the ratio of wall material formulation. Adding WPC to emulsion compounds reduces dents and increases the surface uniformity of microcapsules due to slow drying rate of the wall matrix and creation of elasticity in the wall systems (Simon‐Brown et al., 2015).

The emulsifying property of WPC due to the presence of hydrophobic particles leads to better emulsification at higher amounts of WPC, and WGE can disperse finer, resulting in smaller size and better dispersion of emulsion particles (Basak et al., 2015). Since the initial freezing prior to freeze‐drying aggregates and attaches the emulsion droplets together, the microcapsules obtained by this method are also larger in size (Desobry et al., 1997).

Anwar and Kunz (2011) presented evidence that microcapsules obtained by freeze drying had an irregular, very light, and porous structure compared to those produced by spray drying. The occurrence of unstable phenomena, such as droplet aggregation, interconnection, and mixing during storage, also increases the particle diameter (Dickinson, 2003). Zhang et al. (2019) showed modification methods of wheat bran insoluble dietary fiber contributed to alteration in morphology of that.

### 3.7 Effect of drying method and coating material on DPPH free radical scavenging activity of microencapsulated WGE

**FIGURE 6:**
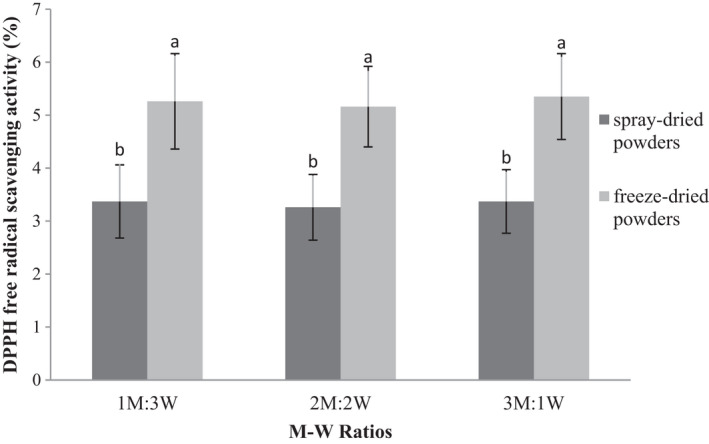
Antioxidant activity of encapsulated wheat germ extract at different ratios of maltodextrin to whey protein concentrate (M‐W)

An increase in the M‐W ratio had no significant effects on the DPPH free radical scavenging activity of produced powders (*p* ≤ .05), while drying method had a significant effect on the DPPH free radical scavenging activity of obtained powders (*p* ≤ .05) (Figure 6). Drying agents in spray dryers better protect phenolic compounds than freeze dryers during drying process, which increase DPPH free radical scavenging activity in spray‐dried powders that generally contained higher phenolic compounds (Simon‐Brown et al., 2015). Moreover, water activity, storage temperature, and artificial light were reported to affect the phenolic content and anthocyanin content of produced bayberry microcapsules and degraded phenolic compounds (Fang & Bhandari, 2011).

The type of wall material can play an important role in the oxidative stability of the product. Therefore, given the limitation of maltodextrin in the emulsification process and the effective role of emulsion properties on the properties of produced powders, it is concluded that the addition of WPC had a considerable effect on lowering the oxidation of microcapsules. The simultaneous presence of carbohydrates and proteins in the wall structure improve the emulsifying properties and oxidative stability of product (Yoshii et al., 2001). Enhanced oxidation stability of microencapsulated components has been reported in previous studies. Microencapsulation of flaxseed oil by spray drying led to increased oxidative stability in this oil (Carneiro et al., 2013). Similarly, DPPH free radical scavenging activity of ginger extract microcapsules obtained by spray drying was not significantly different between microcapsules studied at different ratios of maltodextrin and gum arabic (Simon‐Brown et al., 2015).

### 3.8 Effect of drying method and coating material on lipase activity of microencapsulated WGE

**FIGURE 7:**
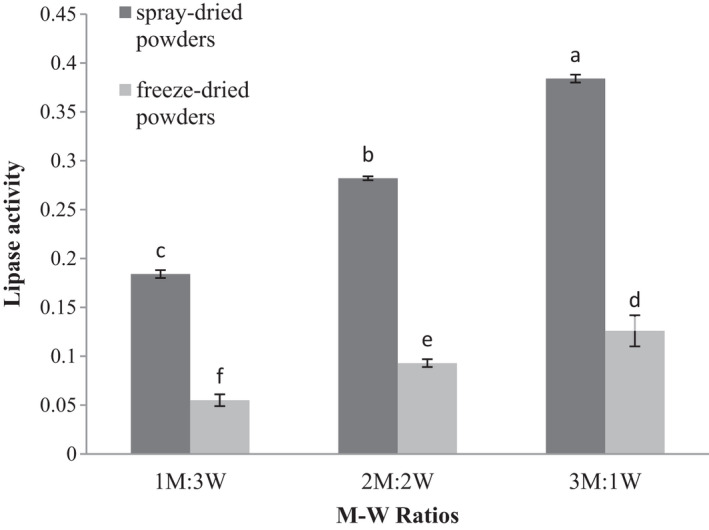
Lipase activity (μmol para nitrophenol/min) of encapsulated wheat germ extract at different ratios of maltodextrin to whey protein concentrate (M‐W)

The M‐W ratio and drying methods significantly influenced lipase activity (*p* ≤ .05), an increase in the M‐W ratio leading to elevated lipase activity in the powders produced from spray and freeze dryers. The highest and the lowest lipase activities were found in M‐W ratios of 3:1 and 1:3, respectively (Figure 7). An elevation in maltodextrin concentration results in the formation of a denser network structure in the microcapsules with more coherent pores and thus entrapment of extra enzymes (Soliman et al., 2013).

Lipase activity in spray‐dried powders in all M‐W ratios was higher than that of freeze‐dried powders (Figure 7). Cold shock resulting from initial freezing in the freeze‐drying method is likely to alter the enzyme structure possibly resulting in inactivity or decreased biological activity of the enzyme. A research demonstrated that changes in inlet and outlet air temperatures and low flow rate in spray drying caused the loss of lipase enzyme activity (Andrea et al., 2016). Truong and Wunwisa (2016) microencapsulated microbial protease and lipase using alginate and various types of copolymers such as maltodextrin, xanthan, and chitosan. They noticed that the enzyme activity was maintained during storage and the leakage of these two enzymes decreased by increasing concentrations of wall materials (alginate and copolymer).

### 3.9 Effect of drying method and coating material on acid phosphatase activity of microencapsulated WGE

**FIGURE 8:**
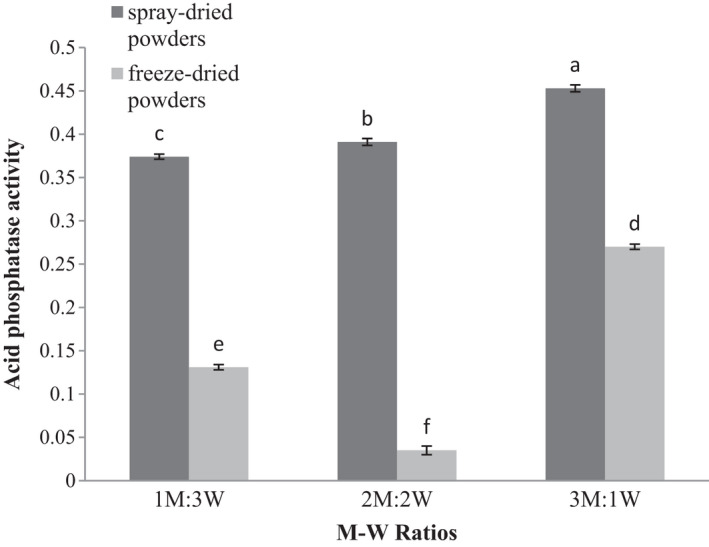
acid phosphatase activity (μmol glycerol/min) of encapsulated wheat germ extract at different ratios of maltodextrin to whey protein concentrate (M‐W)

The M‐W ratio and drying methods significantly impacted acid phosphatase activity (*p* ≤ .05). Increase in the M‐W ratio, leading to elevated enzyme activity in the powders produced by both spray and freeze dryers. The highest and the lowest acid phosphatase activities were found in M‐W ratios of 3:1 in spray‐dried powders and M‐W ratios of 2:2 in freeze‐dried powders, respectively (Figure 8). Unstable cross‐links between wall materials and ions, short‐time dispersion of microcapsules, and poor mechanical stability of the formed membrane may result in high enzyme leakage and declined activity in a 2:2 (M‐W) ratio in freeze‐dried powders (Siti‐ Noraida et al., 2013). The activity of acid phosphatase in spray‐dried powders in all M‐W ratios was higher than that of freeze‐dried powders (Figure 8), which is similar to that observed for lipase activity.

### 3.10 Effect of drying method and coating material on enzyme microencapsulation efficiency of microencapsulated WGE

**FIGURE 9:**
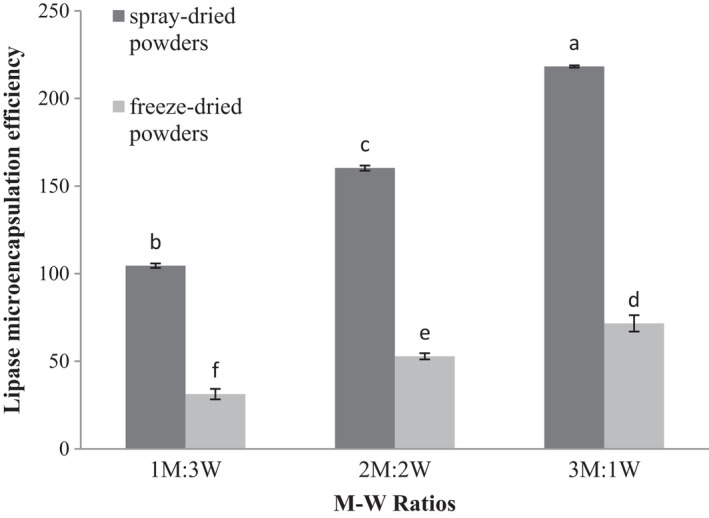
Lipase microencapsulation efficiency of encapsulated wheat germ extract at different ratios of maltodextrin to whey protein concentrate (M‐W)

Increase in the M‐W ratio lead to increased lipase efficiency in the powders produced by spray and freeze dryers (*p* ≤ .05). The highest and the lowest lipase microencapsulation efficiency were found in M‐W ratios of 3:1 and 1:3, respectively (Figure 9). In spray‐dried powders, an increase in maltodextrin concentration resulted in uniform surfaces of microcapsules and decreased surface cracks, thereby reducing lipase leakage and elevating lipase microencapsulation efficiency (Basak et al., 2015).

Increase in the M‐W ratio led to increased amount of lipase microencapsulation efficiency in freeze‐dried powders. The microencapsulation efficiency of lipase was higher in spray drying than in freeze drying method (Figure 9). The uniform particle size and spherical particle shape in spray drying method increased the microencapsulation efficiency of lipase compared to freeze drying method. However, WPC as an emulsifying and layer forming agent, as well as good coating strength, resulted in rapid formation of a semipermeable layer on the surface of microcapsules, which prevented the enzyme leakage and increased the microencapsulation efficiency of lipase (Thijssen & Rulkens, 1968).

Simon‐Brown et al. (2015) microencapsulated ginger extract with spray drying method at different ratios of maltodextrin and gum arabic as wall materials. Surface cracks and dents were lower in produced microcapsules containing higher ratios of maltodextrin, with better protection of microencapsulated bioactive compounds.

**FIGURE 10:**
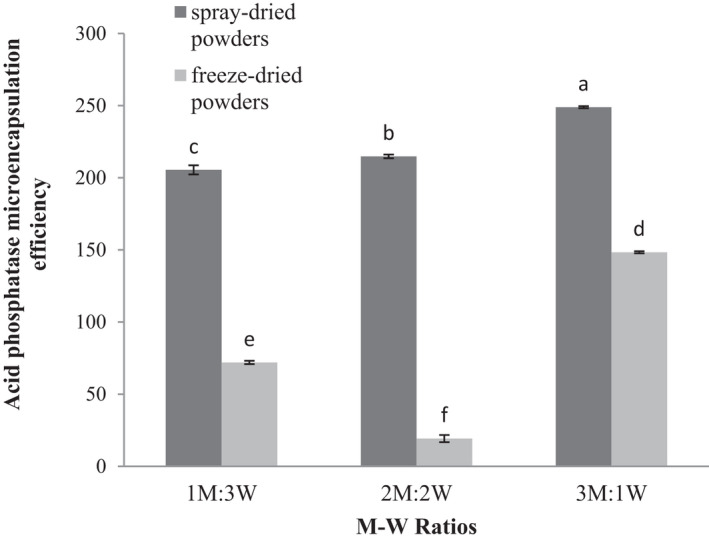
Acid phosphatase microencapsulation efficiency of encapsulated wheat germ extract at different ratios of maltodextrin to whey protein concentrate (M‐W)

Increase in the M‐W ratio and drying methods significantly impacted microencapsulation efficiency of acid phosphatase in the produced powders (*p* ≤ .05), resulting in elevated enzyme efficiency in the powders produced by both spray and freeze dryers. The uppermost and the lowermost microencapsulation efficiency of acid phosphatase were recorded in M‐W ratios of 3:1 in spray‐dried powders and 2:2 in freeze‐dried powders, respectively. The microencapsulation efficiency of acid phosphatase was higher in spray drying than in freeze drying method. The results are similar to those obtained for the microencapsulation efficiency of lipase (Figure 10).

## 4 CONCLUSION

Results of this study demonstrate that the amounts of moisture, solubility, bulk density, particle size, morphology of microcapsules, lipase activity, acid phosphatase activity, and microencapsulation efficiency of lipase and acid phosphatase are dependent on the type and concentrations of wall materials as well as the method of microencapsulation. Microencapsulation method had a significant effect on DPPH free radical scavenging activity. An increase in the M‐W ratio led to decreased moisture content, but it elevated solubility, lipase activity, acid phosphatase activity, and microencapsulation efficiency of lipase and acid phosphatase in both drying methods. In the spray drying method, particle size and bulk density increased with rising M‐W ratio, whereas these two parameters decreased in the freezing method. A comparison between the microencapsulation methods revealed that the spray drying method resulted in improvements of moisture, solubility, DPPH free radical scavenging activity, lipase activity, acid phosphatase activity, and microencapsulation efficiency of lipase and acid phosphatase, as well as dropped particle size. Bioactive compounds, lipase and acid phosphatase enzymes present in WGE, were reduced during the process and storage, and microencapsulation process enables the protection of bioactive components of that. The encapsulated WGE is in a powder form could be readily incorporated into food products such as dairy products.

## 5 INFORMED CONSENT

Not applicable.

## CONFLICT OF INTEREST

The authors declare that they have no conflict of interest.

## ETHICAL APPROVAL

This article does not contain any studies with animals performed by any of the authors.
